# Single Sporadic Deceleration during Reactive Nonstress Test—Clinical Significance and Risk for Cesarean Delivery

**DOI:** 10.3390/jcm12103387

**Published:** 2023-05-10

**Authors:** Hila Weinberger, Shlomit Nekave, Mordechai Hallak, Amir Naeh, Rinat Gabbay-Benziv

**Affiliations:** 1 Obstetrics and Gynecology Department, Hillel Yaffe Medical Center, Hadera 38100, Israel; hiladav@gmail.com (H.W.); mottih@hy.health.gov.il (M.H.); gabbayrinat@gmail.com (R.G.-B.); 2 The Rappaport Faculty of Medicine, Technion—Israel Institute of Technology, Haifa 32000, Israel; shlomitnekave@gmail.com

**Keywords:** fetal heart rate monitoring, single sporadic deceleration, cesarean delivery

## Abstract

Objective: Evidence regarding the clinical significance of a single sporadic variable deceleration (SSD) in reactive non-stress test (NST) is scarce, and optimal management has yet to be established. We aim to evaluate whether SSD during a reactive NST at term is associated with a higher risk for fetal heart rate decelerations during labor and the need for intervention. Methods: This was a retrospective, case-control study of singleton term pregnancies at one university-affiliated medical center in 2018. The study group consisted of all pregnancies with an SSD in an otherwise reactive NST. For each case, two consecutive pregnancies without SSD were matched in a 1:2 ratio. The primary outcome was the rate of cesarean delivery (CD) due to non-reassuring fetal heart rate monitoring (NRFHRM). Results: 84 women with an SSD were compared to 168 controls. SSD during antenatal fetal surveillance did not increase the rate of CD overall or for NRFHRM (17.9% vs. 13.7% and 10.7% vs. 7.7%, respectively, *p* > 0.05). Rates of assisted deliveries and maternal and neonatal complications were similar between the groups. Conclusions: SSD during a reactive NST in term pregnancies is not associated with an increased risk for adverse perinatal outcomes. SSD should not necessarily require induction of labor, and expectant management is a reasonable alternative.

## 1. Introduction

Fetal heart rate (FHR) monitoring using cardiotocography is the most widely applied technique for antepartum fetal assessment with the goal of reducing stillbirth [1]. FHR monitoring is based on the premise that a heart rate of a fetus that is not acidotic or neurologically depressed will temporarily accelerate with fetal movements. Moreover, heart rate reactivity is thought to be a good indicator of normal fetal autonomic function [2].

Nonstress test (NST) monitoring refers to cardiotocography without contractions and is routinely used to assess the risk of fetal death in pregnancies complicated by maternal conditions such as diabetes mellitus or fetal conditions such as fetal growth restriction [3]. NST is considered reactive when the cardiotocography demonstrates two or more FHR monitor accelerations within a 20-min period of monitoring [4]. When contractions are present, then monitoring changes to contraction stress test (CST), which evaluates the FHR response to the transient hypoxemic condition caused by contractions (for example, during labor).

The reliability of NST and CST as predictors of fetal status is controversial [1,5]. During labor, the NST false-negative rate is estimated at 0.3%, but with a 55% false-positive rate [6]. Moreover, previous studies showed that the use of the antepartum NST did not reduce rates of perinatal death or neonatal seizures [7].

Variable decelerations are common during FHR monitoring and may be seen in up to 50% of NSTs [8]. The evidence regarding the clinical significance of a single sporadic variable deceleration (SSD) during FHR tracing during reactive NST is scarce, and previous studies have yielded conflicting results [8,9,10,11,12,13,14,15]. Therefore, the optimal management of SSD during NST for a fetus without other signs of fetal compromise has not been established.

Thus, in this study, we aim to evaluate whether SSD during a reactive NST at term is associated with a higher risk for FHR decelerations during labor and cesarean deliveries.

## 2. Materials and Methods

### 2.1. Study Population and Data Retrieval

This was a retrospective, case-control, non-interventional study of all singleton, term pregnancies at one university-affiliated medical center in 2018. The study group consisted of all pregnancies with an SSD in an otherwise reactive NST performed during antenatal fetal surveillance, before any signs of delivery, at 37–41 gestational weeks. The decision to admit the patient for fetal surveillance, induce labor, or discharge the patient for ongoing ambulatory care was taken individually and at the attending physician’s discretion. Patients who were discharged for expectant management were advised for ambulatory weekly NSTs. In cases where it was decided to induce labor, the induction usually started within a few hours.

For each case in the study group, two consecutive pregnancies without SSD were matched by age, parity, gestational age, the onset of labor, and history of previous cesarean delivery (CD) in a 1:2 ratio. Pregnancies complicated by multiple gestations, fetal malformations, oligohydramnios, and suspected fetal growth restriction were excluded. Furthermore, women that were scheduled for elective cesarean section were excluded.

Maternal and neonatal data were retrieved manually by study personnel from the center’s computerized comprehensive perinatal database. Maternal, perinatal, and neonatal data are documented prospectively on admission and immediately after delivery. Retrieved data included maternal characteristics (e.g., age, BMI, parity, and previous medical and obstetrics background). Delivery outcomes included gestational age at delivery, need for induction, mode of delivery, and neonatal and maternal outcomes until discharge.

The primary outcome of the study was defined as the rate of CD due to non-reassuring fetal heart rate monitoring (NRFHRM). Additionally, we compared outcomes, including the presence of FHR decelerations during labor, meconium in labor, and rates of fetal-indicated assisted deliveries. Other maternal complications include postpartum hemorrhage, need for blood transfusion, and maternal hospitalization time, as well as other neonatal outcomes including birthweight, pH, Apgar score, rates of cephalohematoma and hypoglycemia, and need for neonatal intensive care unit (NICU) admission were compared between the groups.

### 2.2. Definitions

Reactive NST was defined as NST with at least two or more FHR accelerations from a normal baseline of 110–160 beats per minute within a 20-min period of monitoring [4]. SSD was defined as a single decrease in FHR of 15 beats per minute or greater, lasting 15 s or greater, and less than 2 min during a 20-min otherwise reactive NST [16]. NRFHRM was defined as fetal heart rate patterns, which were classified as either category II or category III, according to the National Institute of Child Health and Human Development workshop report [17].

Gestational age at delivery was determined by the parturients’ last menstrual period and verified by first-trimester ultrasound, when available. Hypertensive disorders were classified as gestational hypertension or preeclampsia according to conventional criteria.

Large for gestational age (LGA) and small for gestational age (SGA) were defined as birthweight above the 90th percentile or below the 10th percentile, respectively, for a given gestational age, using local population-based curves [18].

### 2.3. Statistical Analysis

Data analysis was performed using SPSS version 21.0 software (SPSS, Inc., Chicago, IL, USA). *p* < 0.05 was considered significant. Continuous variables were compared using Student’s *t*-test and Mann-Whitney U test. The chi-square and Fisher’s exact tests were used for categorical variables, as appropriate. Following univariate analysis, a logistic regression analysis was used to adjust outcomes for potential confounders. Variables with previously known clinical impact or variables that were found significant in the univariate analysis entered the regression.

## 3. Results

Overall, during the study period, there were 4463 deliveries in our center with a CD rate of 20.8% (927/4463). Four hundred and sixteen (44.9%) cesareans were elective CDs, and 55.1% were non-elective. The rate of vacuum-assisted vaginal deliveries (VAVD) was 7.6% (270/3536). 

The study cohort included 84 women with a diagnosis of SSD during reactive NST that were compared to 168 women in the control group (Table 1). The mean maternal age of the cohort was 29.6 years, and 50% of women were nulliparas. Two women in the cohort had pregestational diabetes, and three women suffered from chronic hypertension. Overall, the study group had similar demographic and obstetrical characteristics as the control group.

Pregnancy outcomes are shown in Table 2. For the entire cohort, rates of induction were 76.2% (192/252), and the mean gestational age at delivery was 39.7 ± 1.1 weeks. SSD during antenatal fetal surveillance was not associated with the presence of decelerations or meconium during labor; however, the study group had a higher rate of amnioinfusion use (3/84 vs. 0/168, *p* < 0.05). SSD during antenatal fetal surveillance did not increase the rate of CD overall or for NRFHRM (17.9% vs. 10.7% and 10.7% vs. 7.7%, study vs. control group, respectively). Likewise, rates of VAVD were similar between the groups.

Maternal and neonatal outcomes are detailed in Table 3. Rates of postpartum hemorrhage, obstetric anal sphincter injuries, and blood transfusion were comparable between the groups. Similarly, the incidence of neonatal complications was similar between groups.

Table 4 demonstrates delivery outcomes in the study group, stratified by their management—Induction of labor vs. expectant management. Cases that were managed expectantly did not have an increased rate of FHR declarations during labor, meconium, or CD. Rates of neonatal pH < 7.2 were similar between the groups.

## 4. Discussion

In the present study, we aimed to evaluate the clinical significance of an SSD during antenatal fetal surveillance at term. Our study results demonstrated the following: 1. SSD was not associated with an increased rate of CD overall or for NRFHRM; 2. There were no differences in other maternal or neonatal outcomes; 3. In the SSD group, expectant management did not increase adverse outcomes or CD rates.

The goal of antepartum fetal surveillance is to reduce the risk of stillbirth. Techniques to assess fetal status include maternal perception of fetal movement, NST, CST, biophysical profile, and umbilical artery Doppler velocimetry [2].

NST has been widely used as a primary method of antepartum fetal surveillance for almost half a century. This approach has proved successful in screening large numbers of patients and has certain advantages over OCT [14,19]. The appearance of accelerations during NST is one of the best indicators of fetal well-being [19]. Loss of NST reactivity is most commonly associated with a fetal sleep cycle but can also result from other causes of central nervous system depression, including fetal acidosis [4]. A reactive NST has been connected with a lower incidence of perinatal morbidity and mortality [19]. However, the significance of SSD during an otherwise reactive NST is yet unclear.

Previous studies have investigated the relationship between antepartum decelerations, intrapartum fetal distress, and perinatal outcomes with conflicting results. Meis et al. examined 908 NSTs and showed that brief variable deceleration existed in more than half of them [8]. The authors concluded that isolated variable decelerations were not associated with FHR decelerations during labor, low Apgar scores at birth, or SGA neonates. However, the combination of variable decelerations and changes in variability, or fetal bradycardia, was found to be associated with adverse neonatal outcomes [11,14]. A correlation was also found between variable decelerations and umbilical cord abnormalities [12]. Fujimori et al. investigated the relationship between sporadic FHR decelerations and fetal electrocortical changes in eight physiologically normal sheep fetuses [20]. SSDs were found to be associated with fetal electrocorticogram changes but without significant changes in pH or blood gases.

Most SSDs are most likely variable decelerations that are caused by temporary cord compression [9]. Pressure on the cord initially occludes the umbilical vein, which results in an acceleration and indicates a healthy response. If the pressure continues and increases, it is followed by occlusion of the umbilical artery, which results in a sharp deceleration as the fetal blood supply is suddenly restricted [1]. Initial management of variable decelerations includes the provision of maternal oxygen, change in maternal position, discontinuation of labor stimulation, and treatment of maternal hypotension [17]. Oligohydramnios is associated with more frequent variable decelerations as the amniotic fluid has a protective role on the fetal umbilical cord, and some studies have suggested that the use of amnioinfusion may improve the FHR pattern [21]. Interestingly, in our study, cases with SSD had a significantly higher rate of amnioinfusion during labor, but ultimately the rate of CD was similar between the groups, which still makes it a controversial intervention.

Even though SSDs are common, very few data exist regarding their prevalence and clinical significance. Jaschevatzk et al. examined the significance of SSD in term pregnancies [9]. Out of 4742 NSTs performed, 34 had an SSD and were hospitalized for further monitoring. Of these, 14 patients were found to have recurrent decelerations and had undergone induction of labor. In comparison between patients with recurrent decelerations and patients without SSD, it was found that in the recurrent decelerations group, there was a higher rate of NRFHRM during labor but no differences in meconium, Apgar score, or perinatal mortality rate. Although limited by a small sample size, this study showed no difference in neonatal outcomes, even in cases with recurrent decelerations. Hagay et al. found SSD in 62 of 7202 NSTs between 32–42 weeks [10]. Excluding four cases with positive oxytocin challenge test, the remaining 58 patients were followed as inpatients until spontaneous labor began. Of them, eight had fetal distress during labor, and four of them required a cesarean section. The authors concluded that SSD is associated with a higher incidence of fetal distress during labor. To note, compared to our study, this study differed in gestational age in which SSD was observed, as well as the definition of SSD, which included longer and deeper decelerations.

In cases of SSD in an otherwise reactive NST, especially near term, we find that it is not uncommon for physicians to feel uncomfortable with discharging the patient for an outpatient follow-up, concerned that this may put the fetus at risk. In a sub-analysis of the study group, we demonstrated that expectant management of SSD was not associated with a higher risk for FHR decelerations during labor, meconium, CD for NRFHRM or pH < 7.2, and therefore can be considered as a reasonable and safe alternative for induction of labor.

Our study’s strengths include the meticulous matching between control and study groups and the ethnic diversity of the population admitted and delivered in our institute. Moreover, our study was based on a manual review of our computerized database, which is under constant, prospective, meticulous review by senior attending physicians. Lastly, to the best of our knowledge, this is among the few, if not the only, study that evaluated the clinical significance of SSD in contemporary care. However, the study is not free of limitations, including its retrospective design and limited cohort size in a single medical center, which may be underpowered to evaluate significant maternal and neonatal outcomes.

In conclusion, our study demonstrates that SSD in an otherwise uncomplicated term pregnancy is not associated with FHR decelerations during labor, operative or cesarean delivery, or neonatal complications. Moreover, induction of labor in the study group was not associated with less CD for NRFHRM or with better obstetric outcomes. Therefore, SSD should not necessarily require induction of labor, and expectant management is a reasonable alternative. Larger studies are needed to further investigate the clinical significance of SSD and to evaluate long-term neonatal outcomes.

## Figures and Tables

**Table 1:** Maternal demographics and obstetric characteristics stratified by the presence of a single sporadic deceleration.

	SSD (N = 84)	No SSD (N = 168)	*p*-Value
Maternal age (years)	29.6 ± 5.5	29.7 ± 5.2	0.94
BMI (Kg\m^2^)	24.7 ± 5.4	25.3 ± 5.5	0.44
Ethnicity:			
• Jewish	38 (45.2)	96 (57.1)	0.083
• Arabic	46 (54.8)	72(42.9)	
Maternal smoking	2 (2.4)	12 (7.3)	0.091
Pregestational diabetes mellitus	1 (1.2)	1 (0.6)	0.55
Chronic hypertension	0 (0)	3 (1.8)	0.55
Nulliparity	42 (50)	84 (50)	>0.99
Previous cesarean delivery	3 (3.6)	6 (3.6)	>0.99
Hypertensive disorders during pregnancy:			
■ Preeclampsia	2 (2.4)	11 (6.5)	0.076
■ Gestational hypertension	2 (2.4)	13 (7.7)	
Gestational diabetes	11 (13.1)	29 (17.3)	0.46

Values are presented as mean ± SD or n (%). SSD—single sporadic deceleration; BMI—body mass index.

**Table 2:** Delivery outcomes stratified by the presence of a single sporadic deceleration.

	SSD (N = 84)	No SSD (N = 168)	*p*-Value
Gestational age at delivery (weeks)	39.8 ± 1.1	39.6 ± 1.1	0.12
Induction of labor	60 (71.4)	132 (78.6)	0.21
FHR decelerations during labor	15 (17.9)	18 (10.7)	0.11
Meconium	18 (21.4)	22 (13.1)	0.10
Amnioinfusion during labor	3 (3.6)	0 (0)	0.03
Fever during labor	0 (0)	14 (8.3)	0.006
Mode of delivery:			
• Spontaneous vaginal delivery	64 (76.1)	137 (81.5)	0.6
• VAVD delivery	5 (6)	8 (4.8)	
• Cesarean delivery	15 (17.9)	23 (13.7)	
Cesarean delivery indication			
• NRFHRM	9 (60)	13 (52.2)	0.59
• Arrested labor	6 (40)	9 (39.1)	
• Other	0 (0)	1 (4.3)	
Cesarean delivery for NRFHRM	9 (10.7)	13 (7.7)	0.48
VAVD indication			
■ NRFHRM	3 (60)	6 (75)	0.51
■ Arrested labor	2 (40)	2 (25)	

Values are presented as mean ± SD or n (%). SSD—single sporadic deceleration; VAVD—vacuum-assisted vaginal delivery; FHR—fetal heart rate; NRFHRM—non-reassuring fetal heart rate monitoring.

**Table 3:** Maternal and neonatal outcomes stratified by the presence of a single sporadic deceleration.

	SSD (N = 84)	No SSD (N = 168)	*p*-Value
Maternal outcomes:			
Postpartum hemorrhage	4 (4.8)	4 (2.4)	0.44
OASIS	1 (1.2)	2 (1.2)	>0.99
Blood transfusion	0 (0)	6 (3.5)	0.08
Neonatal outcomes:			
Birthweight (grams)	3302 ± 393	3313 ± 392	0.91
Male gender	43 (51.2)	92 (54.8)	0.5
PH < 7.2	11 (14.1)	14 (8.9)	0.26
Apgar 5 min < 7	1 (1.2)	4 (2.4)	0.46
NICU admission	2 (2.4)	1 (0.6)	0.25
Cephalohematoma	1 (1.2)	0 (0)	0.33
Hypoglycemia	1 (1.2)	0 (0)	0.33
Asphyxia	1 (1.2)	0 (0)	0.33

Values are presented as mean ± SD or n (%). SSD—single sporadic deceleration; OASIS—Obstetric anal sphincter injuries.

**Table 4:** Delivery outcomes, Induction of labor vs. expectant management in the single sporadic deceleration group.

	Induction of Labor (N = 60)	Expectant Management (N = 24)	*p*-Value
FHR decelerations during labor	12 (20)	3 (12.5)	0.31
Meconium	11 (18.3)	7 (29.1)	0.21
Amnioinfusion during labor	3 (5)	0 (0)	0.35
Mode of delivery:			
• Spontaneous vaginal delivery	44 (73.3)	20 (83.3)	0.6
• VAVD delivery	4 (6.6)	1 (4.1)	
• Cesarean delivery	12 (20)	3 (12.5)	
Cesarean delivery for NRFHRM	7 (58.3)	2 (66.6)	0.49
PH < 7.2	6 (10.9)	5 (21.7)	0.18

Values are presented as n (%). FHR—fetal heart rate; NRFHRM—non-reassuring fetal heart rate monitoring.

## Data Availability

The data presented in this study are available on request from the corresponding author. The data are not publicly available due to privacy and ethical considerations.

## References

[1] Landon M.B., Driscoll D.A., Jauniaux E.R., Galan H.L., Grobman W.A., Berghella V. (2018). Gabbe’s Obstetrics Essentials: Normal & Problem Pregnancies.

[2] American College of Obstetricians and Gynecologists (2021). Antepartum fetal surveillance: ACOG practice bulletin, number 229. Obstet. Gynecol..

[3] American College of Obstetricians and Gynecologists, Committee on Obstetric Practice, Society for Maternal-Fetal Medicine (2021). Indications for outpatient antenatal fetal surveillance: ACOG Committee Opinion, Number 828. Obstet. Gynecol..

[4] Evertson L.R., Gauthier R.J., Schifrin B.S., Paul R.H. (1979). Antepartum fetal heart rate testing: I. Evolution of the nonstress test. Am. J. Obstet. Gynecol..

[5] Nelson K.B., Dambrosia J.M., Ting T.Y., Grether J.K. (1996). Uncertain value of electronic fetal monitoring in predicting cerebral palsy. N. Engl. J. Med..

[6] Signore C., Freeman R.K., Spong C.Y. (2009). Antenatal testing—A reevaluation: Executive summary of a Eunice Kennedy Shriver National Institute of Child Health and Human Development workshop. Obstet. Gynecol..

[7] Pattison N., McCowan L. (1999). Cardiotocography for Antepartum Fetal Assessment.

[8] Meis P.J., Ureda J.R., Swain M., Kelly R.T., Penry M., Sharp P. (1986). Variable decelerations during nonstress tests are not a sign of fetal compromise. Am. J. Obstet. Gynecol..

[9] Jaschevatzky O.E., Marom D., Ostrovsky P., Ellenbogen A., Anderman S., Ballas S. (1998). Significance of sporadic deceleration during antepartum testing in term pregnancies. Am. J. Perinatol..

[10] Hagay Z.J., Mazor M., Leiberman J.R., Katz M., Insler V. (1983). The significance of single sporadic deceleration during a nonstress test. Eur. J. Obstet. Gynecol. Reprod. Biol..

[11] Anyaegbunam A., Brustman L., Divon M., Langer O. (1986). The significance of antepartum variable decelerations. Am. J. Obstet. Gynecol..

[12] O’Leary J.A., Andrinopoulos G.C., Giordano P.C. (1980). Variable decelerations and the nonstress test: An indication of cord compromise. Am. J. Obstet. Gynecol..

[13] Bourgeois F.J., Thiagarajah S., Harbert G.M. (1984). The significance of fetal heart rate decelerations during nonstress testing. Am. J. Obstet. Gynecol..

[14] Druzin M.L., Gratacós J., Keegan K.A., Paul R.H. (1981). Antepartum fetal heart rate testing: VII. The significance of fetal bradycardia. Am. J. Obstet. Gynecol..

[15] Pazos R., Vuolo K., Aladjem S., Lueck J., Anderson C. (1982). Association of spontaneous fetal heart rate decelerations during antepartum nonstress testing and intrauterine growth retardation. Am. J. Obstet. Gynecol..

[16] Arnold K.C., Flint C.J. (2017). Intrapartum fetal heart rate monitoring: Nomenclature, interpretation, and general management principles. Obstetrics Essentials.

[17] Macones G.A. (2009). The 2008 National Institute of Child Health and Human Development Workshop Report on Electronic Fetal Monitoring: Update on Definitions, Interpretation, and Research Guidelines. Obstet. Gynecol..

[18] Barth W.H., Jackson R. (2020). Macrosomia ACOG Practice Bulletin, Number 216. Obstet. Gynecol..

[19] Clark S.L., Sabey P., Jolley K. (1989). Nonstress testing with acoustic stimulation and amniotic fluid volume assessment: 5973 tests without unexpected fetal death. Am. J. Obstet. Gynecol..

[20] Fujimori K., Murata Y., Sato A. (2006). Sporadic fetal heart rate decelerations associated with electrocortical changes in fetal lambs. J. Obstet. Gynaecol. Res..

[21] Katsura D., Takahashi Y., Iwagaki S., Chiaki R., Asai K., Koike M., Nagai R., Yasumi S., Furuhashi M. (2019). Amnioinfusion for variable decelerations caused by umbilical cord compression without oligohydramnios but with the sandwich sign as an early marker of deterioration. J. Obstet. Gynaecol..

